# The Dynamic Changes of Circulating Myeloid-Derived Suppressor Cells (MDSCs) Subsets in Colorectal Cancer Patients Undergoing Oxaliplatin-Based Chemotherapy

**DOI:** 10.1007/s12029-025-01207-x

**Published:** 2025-03-26

**Authors:** Ralph Girson Gunarsa, Aru Wisaksono Sudoyo, Ricci Steven, Dilafitria Fauza, Fajar Lamhot Gultom, Ibrahim Basir, Dicky Kurniawan, Akterono Dwi Budiyati

**Affiliations:** 1 MRCCC Siloam Hospitals Semanggi, Jakarta, Indonesia; 2 Stem Cell and Cancer Institute, Jalan Jenderal Ahmad Yani No.2, Pulo Gadung, Jakarta, Indonesia

**Keywords:** Colorectal, Cancer, Myeloid-derived suppressor cells, Chemotherapy

## Abstract

**Purpose:**

Increased level of circulating myeloid -derived suppressor cells (MDSCs) in colorectal cancer (CRC) patients has been associated with higher tumor stage and poorer survival due to poorer response to therapeutic agents. However, studies reported inconsistent results on how chemotherapeutic agents affecting the depletion of two major types of MDSCs, polymophonuclear MDSC (PMN-MDSC) and monocytic MDSC (M-MDSC). The present study aims to learn deeper on the dynamic changes of circulating MDSCs, especially in response to oxaliplatin-based treatment in CRC patients.

**Methods:**

This was a prospective study that recruited 30 treatment-naive patients with varying stages of CRC who were scheduled to receive oxaliplatin-based chemotherapy. Blood sampling was conducted prior to and at several time points during and after chemotherapy. Multicolor flow cytometry assay was used to analyse the proportion of HLA-DR^–^ and CD33^+^ with CD15^+^ (PMN-MDSCs) or CD14^+^ (M-MDSCs) cells within peripheral blood mononuclear cells (PBMCs). Other essential tumor biomarkers such as carcinoembryonic antigen (CEA) and tumor-infiltrating lymphocytes (TILs) were also assessed. As a control, 14 healthy subjects were recruited in this study.

**Results:**

Indonesian treatment-naive CRC patients exhibited significantly higher circulating PMN-MDSCs compared to healthy subjects (*p* = 0.003), while M-MDSCs levels showed no significant difference between the groups (*p* = 0.890). Following chemotherapy, the MDSCs level demonstrated dynamic changes. Interestingly, a subgroup of CRC patients with decreased in both PMN- and M-MDSCs levels on D-14 of chemotherapy consistently showed a significant reduction in MDSCs levels during and after therapy completion compared to baseline (*p* = 0.0078).

**Conclusions:**

Circulating MDSCs level, particularly PMN-MDSCs, in CRC patients, was significantly higher compared to healthy subjects. Changes in both circulating PMN- and M-MDSCs levels at D-14 chemotherapy might have prognostic value in oxaliplatin-based chemotherapy.

**Supplementary Information:**

The online version contains supplementary material available at 10.1007/s12029-025-01207-x.

## Introduction

Colorectal cancer (CRC) is the third common cancer occurred in the world that is estimated to have more than 1.9 million new cases and causing more than 900.000 deaths in 2020 [1]. The survival prognosis of CRC decreased sharply as the stage advanced, and unfortunately, there were approximately 25% of newly diagnosed CRC are found in the advanced stage [2]. Although significant improvements in CRC therapy for high-risk and advanced CRC were made in recent years, 25–50% of the cases still develop metastases during disease progression, thus lowering the survival rate [2]. Currently, combinations of chemotherapeutic agents are frequently used as front-liners to treat advanced, high-risk, or metastatic CRC. The most common initial systemic therapy administration involves the combination of fluorouracil (5-FU) and folinic acid (leucovorin), with oxaliplatin (FOLFOX) or irinotecan (FOLFIRI) [3, 4]. However, a recent study demonstrated that resistance to chemotherapy remains deadlock for the disease-free progression and has lowered the survival rates in CRC patients [5].

Myeloid-derived suppressor cells (MDSCs) constitute an immature population of myeloid cells with strong immunosuppressive effects. This population allows inhibition of proliferation and activation of TILs, induction of oxidative stress that causes desensitization of the T cell receptor, and altering macrophage phenotype toward a regulatory phenotype [5, 6]. Evidence from CRC studies suggested that MDSCs, as part of the host immune response, play an important role in disease progression [6, 7]. Their accumulation within the tumor microenvironment (TME) or peripheral blood as circulating MDSCs has influenced CRC progression through stimulation of tumor neovasculogenesis and facilitating local immunosuppression by inhibiting T cell proliferation through secretion of arginase 1 (ARG1) and inducible nitric oxide synthase (iNOS) [5]. Moreover, numerous studies have associated the increased MDSCs level with poorer responses to CRC therapeutic agents, and consequently, MDSCs have been included as therapy targets and important prognostic values in CRC therapy management [8, 9].

In humans, MDSCs are not well characterized. Typically, these cells express the common myeloid markers, CD33, and lack of mature myeloid markers such as HLA-DR [10, 11]. Among MDSC cells, there are two main MDSC subsets associated with tumor progression in CRC, the monocytic (M-) MDSCs expressing CD14^+^ and the granulocytic or polymorphonuclear (PMN-) MDSCs expressing CD15^+^. Both subsets were commonly found to accumulate in peripheral blood of CRC patients and were found to have immunosuppressive effects to cellular anti-tumor immunity response [9–11]. Tumor-mediated PMN-MDSC mainly inhibited T cell function via reactive oxygen species (ROS) through cell-to-cell contact, whereas M-MDSC inhibited T cell mainly through arginase and iNOS. Moreover, T cell inhibition could also be achieved by MDSCs through crosstalk with T regulatory cells (Tregs) via IL-10 and TGF-β cytokines production [10].

Several chemotherapeutic agents used in conventional cancer chemotherapy have been found to reduce MDSC numbers in tumor tissues as well as in the peripheral lymphoid organs [12]. It was reported that FOLFOX, the common first-line chemotherapy, mediated MDSCs depletion via induction of cell death [13]. However, another study demonstrated that FOLFOX-related MDSCs reduction was only observed on the PMN-MDSCs subset, preferentially in patients with high PMN-MDSCs (> 1% in leucocytes) [14]. With regard to these results, the present study aims to evaluate more detailed dynamic changes of circulating M- and PMN-MDSCs in response to oxaliplatin-based treatment in CRC patients. Additionally, circulating MDSCs level was compared to plasma carcinoembryonic antigen (CEA) level as a standard biomarker for CRC surveillance [15], as well as other essential tumor biomarkers. The findings in this study might have important implications for CRC treatment and therapy management.

## Methods

### Patients and Study Design

This was a prospective study aimed at evaluating the MDSCs profile of CRC patients who visited an oncology clinic at the MRCCC Siloam Hospital, Jakarta, Indonesia, in response to oxaliplatin-based chemotherapy. The study was approved by the Medical Ethical Committee of the MRCCC Siloam Hospital (2105/SS/Dir/XII/2020) and performed in accordance with the principles of the revised Declaration of Helsinki. All participants provided written informed consent prior to enrolment.

Eligible patients were of age over 18 years old, naive to any cancer systemic medication, and proposed to receive chemotherapy as first-line therapy following the diagnostics. Tumors were staged according to guidelines [15]. Patients’ eligibility to receive any of oxaliplatin-based therapy: a combination of folinic acid, fluorouracil, and oxaliplatin (FOLFOX), or a combination of capecitabin and oxaliplatin (CAPEOX), or a combination of FOLFOX and irinotecan (FOLFOXIRI) was decided by an oncologist. FOLFOX and FOLFOXIRI were administered on a biweekly basis for a total of 12 cycles while CAPEOX was administered on a 3-weekly basis for a total of 8 cycles.

As a comparison for the MDSC profile baseline, healthy volunteers were recruited from Stem Cell and Cancer Institute (Jakarta, Indonesia) staff with age groups corresponding to the age group of CRC patients.

Prior to therapy, tumor tissue in the formalin-fixed paraffin-embedded (FFPE) block of each CRC patient was collected and characterized using a diagnostic panel consisting of RAS gene family and BRAF mutational status, microsatellite instability (MSI), and tumor-infiltrating lymphocytes (TILs) scoring. MDSCs profile was evaluated along with carcinoembryonic antigen (CEA) level in patients’ blood on the following time points: baseline (before chemotherapy), day 14 (D-14), 25%, 50%, 75%, and 100% of therapy then continued at 3 up to 9 months after therapy ends.

### Specimen Collection and Handling

The FFPE blocks from each patient were provided by the pathology anatomy laboratory of MRCCC Siloam Hospital, Jakarta, Indonesia, or from other hospitals according to the patients’ first biopsy occurrence. The FFPE blocks were then stored in a container and kept in dry and cool storage at room temperature until further processed. Blood samples were collected using an EDTA tube (BD 367856 BD Vacutainer™) and clot-activator tube (BD 366668 BD Vacutainer™) for MDSCs and CEA analysis, respectively. They were stored in a 4 °C refrigerator and analysed within 24 h.

### Tumor Tissue Profiling

All tumor tissue profiling assessment were conducted by Kalgen Innolab Clinical Laboratory (Jakarta, Indonesia). A total of seven slides were obtained from each FFPE block. One of them was processed for hematoxylin and eosin (H&E) staining in order to mark the corresponding tumor-enriched areas of the other one unstained slide and was scratched to obtain DNA from tumor cells. The paraffin flakes were deparaffinized using xylene and centrifuged to pellet the tissue. Following two times washing step in 70% alcohol, the pellets then was proceeded for DNA extraction using QIAamp DNA FFPE Tissue Kit (Qiagen, Germany) according to the manufacturer’s protocol. The extracted DNA was then subjected to PCR-based, high-resolution melting (HRM) protocol to detect mutation that occurred in exons 2, 3, and 4 for both KRAS and NRAS genes, as well as in exon 15 for BRAF gene, as described previously [16].

DNA mismatch repair (MMR) for microsatellite instability (MSI) analysis and TIL scoring were conducted using an immunohistochemistry-based procedure. The primary antibody dilution used was 1:300 followed by overnight incubation. Rabbit XP® was then used as a secondary antibody followed by counterstaining with hematoxylin and slide dehydration. The MMR was determined using rabbit monoclonal antibodies against 4 mismatch repair protein, namely MLH1, MSH2, MSH6, and PMS2 (Agilent, USA) while the TILs scoring using CD3-epsilon (D7A6E) XP (R) Rabbit mAb and CD8-alpha (D8A8Y) Rabbit mAb (Cell Signaling Technology, USA). TIL assessment requires tissue area at least 1.5–2 cm in size including invasive margin (IM) and center of tumor (CT). The center of the tumor is defined as the middle area of the tumor mass, whereas the invasive margin is the border area, with an extent of 1 mm wide, where the tumor cells are directly in contact with surrounding healthy tissue. The invasive margin is located around the edge of tumor tissue that separates it from healthy cells (Appendix Fig. A1). The imaging was performed using a Panoramic scanner (3dHistech, Hungary) followed by digital analysis using pathology software (QuPath, UK). The scoring was achieved by determining the density of CD3 and CD8 T-cells in CT and IM. TIL density was then classified into scores ranging from 0 to 4, in which 0 was valued as low and 4 was valued as high according to density cut off value [17].

### Flow Cytometry

Several cell surface markers were used to identify subsets of MDSCs population based on previous publication [14]. Anti-CD14 (fluorescein isothiocyanate (FITC)-labeled antibody), anti-CD33 (phycoerythrin (PE)-labeled antibody), anti-HLA-DR (peridinin chlorophyll (PerCP)-Cyanine 5.5-labeled antibody), and FITC-labeled anti-CD15 were used to classify MDSC subsets along with appropriate isotype control for each immunoglobulin (BD Bioscience, USA). In brief, two sets of 100 ul of blood samples were incubated with a designated antibody cocktail for 20 min at room temperature. This step was followed by red blood cell lysis using lysing solution (BD Biosciences, USA) and another incubation for 10 min followed by centrifugation (800 × *g*, 5 min). The remaining cells were then washed with staining buffer (1% FBS in PBS KCl), centrifuged at 800 × *g*, 5 min, and finally diluted in flow cytometry buffer (BD Biosciences, USA). The MDSCs were gated based on CD33^+^ and HLA-DR^–^ phenotype and further classified as PMN-MDSC based on CD15^+^ cells or M-MDSC based on CD14^+^ cells (Appendix Fig. A2). Flowcytometric data acquisition was performed using BD FACS Calibur (BD Bioscience, USA), and data analysis was performed using Cell Quest software.

### CEA Measurement

CEA measurement in the serum was conducted at Kalgen Innolab Clinical Laboratory (Jakarta, Indonesia) facility using enzyme-linked fluorescent assay (VIDAS® CEA (S), Biomerieux, USA) according to the manufacturer’s protocol.

### Statistical Analysis

Comparison between different groups were analysed using the Mann–Whitney test, and comparison between continuous data (paired) were analysed using the Wilcoxon test. The data are represented in the median ± interquartile range. Analyses were performed using GraphPad Prism 9.0 software (GraphPad Software Inc., USA).

##  Results

**Fig. 1:**
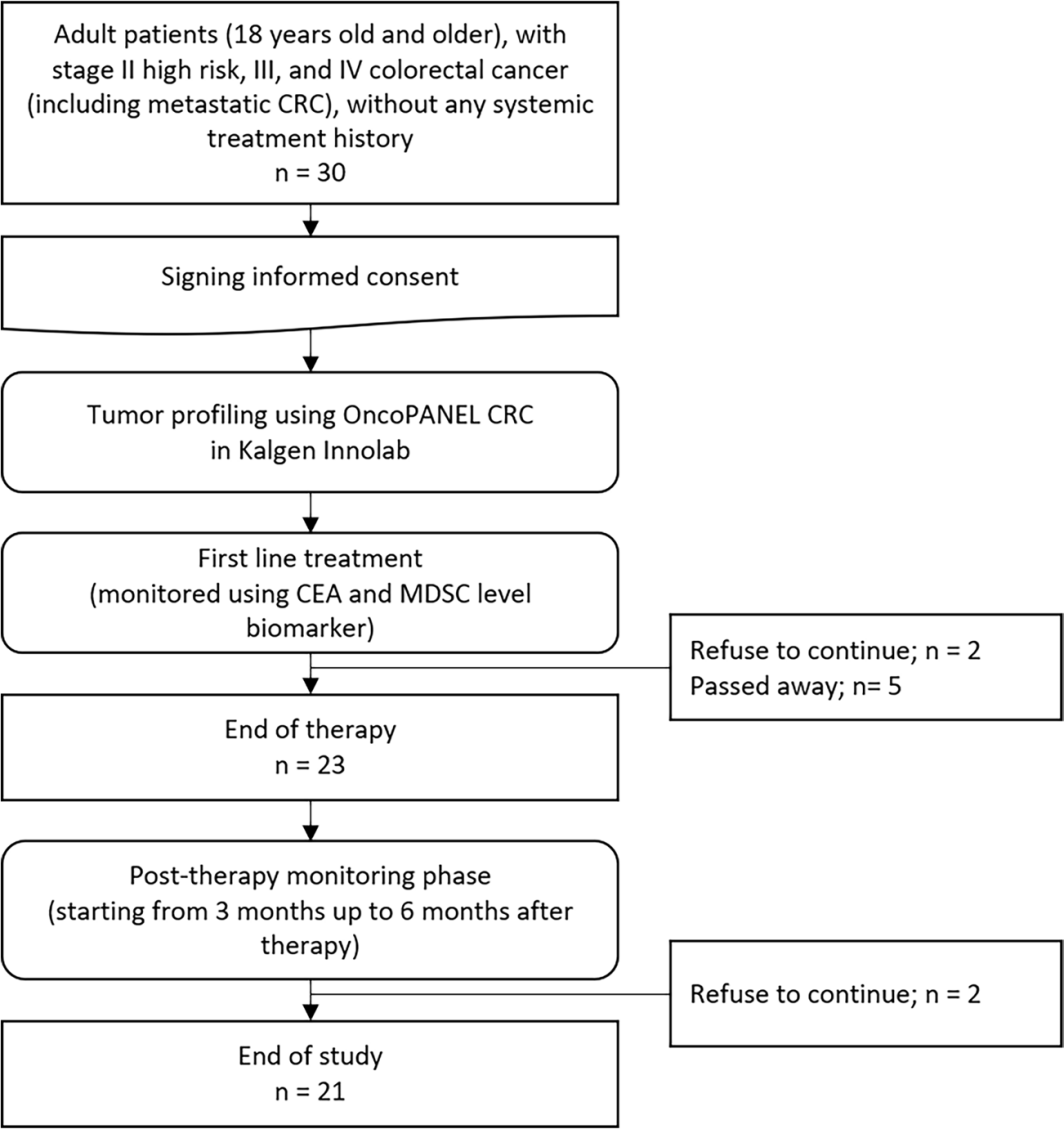
Schematic diagram of patients’ recruitment and assessment

A total of 30 adult CRC patients were recruited to our study between February 2021 and February 2022 at MRCCC Siloam Hospital. During treatment, two patients refused to continue after six cycles of chemotherapy and five patients have been declared passed away. The remaining participants continued the monitoring period starting from 3 months up to 9 months after therapy ended. Among the chemotherapy finishers, three of the patients decided to not participate in the monitoring period leaving a total of 21 patients who made it to the end of the study (Fig. 1).

**Fig. 2:**
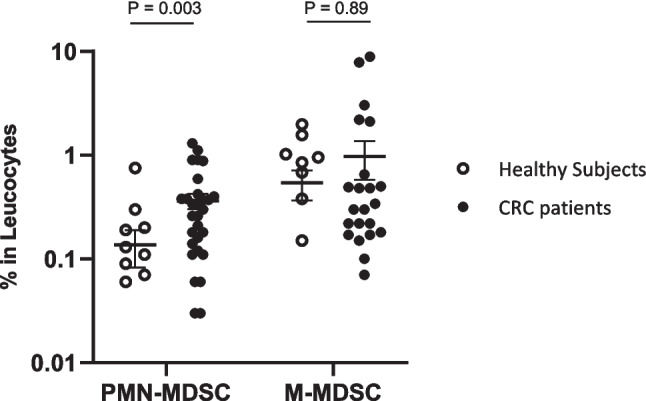
Accumulation of MDSCs in treatment-naive CRC patients compared to healthy donors. The data is presented in percentage and median of each subpopulation. *p*-value was achieved based on the *T*-test

The analysis on the MDSCs baseline profile was conducted on all recruited CRC patients (*n* = 30) and healthy subjects (*n* = 14). The results demonstrated significantly higher proportion of circulating PMN-MDSCs in CRC patients (0.28% ± 0.29) compared to healthy subjects (0.08% ± 0.19) (*p* = 0.003), while the proportion of circulating M-MDSCs in both groups were found to have insignificant difference (0.22% ± 0.49 for CRC patients vs healthy subjects, 0.26% ± 0.97, *p* = 0.89) (Fig. 2) (Appendix Table 1).

Table 1 showed that the patients were 60% (13/21) male with a median of age 58 years old. Eighty-six percent (18/21) of the patients were at advanced tumor stage while the remaining were at stage II high-risk. The tumor characteristics of patients were predominantly consisting of BRAF wildtype (100%), microsatellite stable (MSS) (90%), and high TILs score (80%) while RAS mutant and wildtype showed similar proportion (52% and 48%, respectively). Fourteen out of 21 (67%) patients were treated with FOLFOX, and the other 7 were given either CAPEOX or FOLFOXIRI.

**Table 1:** Summary of patients’ demographic and tumor profile

Total *N* = 21			
**Gender, *n* (%)**		**Treatment, *n* (%)**	
Female	8 (40%)	** CAPEOX**	5 (23.8%)
Male	13 (60%)	** FOLFOX**	14 (66.7%)
Age, median ± interquartile range	58 ± 16.5 years	** FOLFOX w/bevacizumab**	1 (4.75%)
Cancer stage, ***n*** (%)		** FOLFOXIRI**	1 (4.75%)
2a	2 (9.5%)	**RAS, *n* (%)**	
2b	1 (4.7%)	** Mutation**	11 (52.4%)
3	13 (62%)	** Wild type**	10 (47.6%)
4	5 (23.8%)	**BRAF, *n* (%)**	
Metastasis, ***n*** (%)		** Mutation**	0 (0.0%)
Peritoneum	1 (20.0%)	** Wild type**	21 (100%)
Liver	3 (60.0%)	**Microsatellites, *n* (%)**	
Liver, lung, bone	1 (20.0%)	** Instable**	2 (10.0%)
		** Stable**	19 (90.0%)
		**TILs score, *n* (%)**	
		** Intermediate**	4 (20.0%)
		** High**	17 (80.0%)

**Fig. 3:**
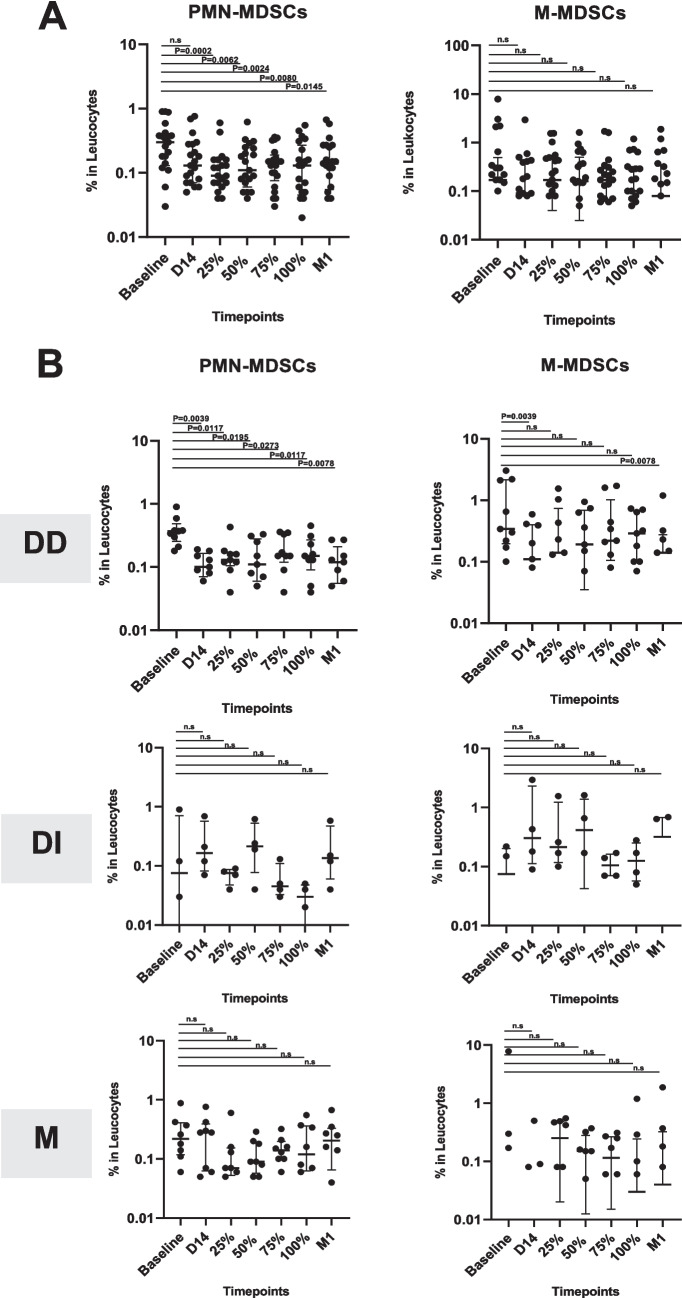
Circulating PMN and M-MDSC levels during and after chemotherapy based on overall analysis (**A**) and stratified to D-14 response (**B**). The data is represented as the median of the log percentage MDSC within the PBMC population. DD = double decreased, meaning there was a decreased level of MDSCs in both PMN- and M-MDSCs at D-14; DI = double increased, meaning there was an increased level of MDSCs in both PMN- and M-MDSCs at D-14; M = increased/decreased level of PMN- and M-MDSCs at D-14 in a mixed manner. n.s, not significant

The analysis based on MDSC subsets revealed that there was a decrease in PMN-MDSCs proportion following chemotherapy initiation at D-14 (0.30% at baseline vs 0.13% at D-14, *p* = 0.097) and consistently lower than baseline at 25% (0.09%, *p* = 0.0002); 50% (0.11%, *p* = 0.0062); 75% (0.15%, *p* = 0.0004); 100% (0.13%, *p* = 0.001) of therapy, and finally at post-therapy period follow up (0.14%, *p* = 0.001) (Fig. 3A). On the other hand, the proportion of M-MDSCs subset was relatively stable during the study. Although we observed changes in the median of M-MDSCs proportion at D-14 and during chemotherapy treatment, as well as at post therapy follow up, however, the Wilcoxon paired-test results showed no significant differences compared to the M-MDSCs at the baseline level (Appendix Table A2).

Further stratification was made according to circulating MDSC data collected on day 14 (D-14) after chemotherapy administration. As described in Fig. 3B, the patients were then divided into 3 subgroups. The first group was named DD group after ‘double-decreased’, which refers to a group of patients with lower circulating both PMN- and M-MDSC levels on D-14 after chemotherapy compared to the baseline (*n* = 9). The second group was named DI or ‘double-increased’, which refers to a group of patients with higher circulating both PMN- and M-MDSC levels on D-14 after chemotherapy compared to the baseline (*n* = 4). Lastly, the third group was named M after ‘mixed’, which refers to a group of patients with only one MDSC subset, either PMN- or M-MDSC, increased on D-14 after the administration of chemotherapy (*n* = 8). A previous study by Limagne et al. in 2016 showed that the circulating MDSCs accumulation in metastatic CRC patients was observed to be decreased 15 days after chemotherapy with FOLFOX-Bevacizumab, which supports the data collection time point and data stratification of the present study (D-14 after chemotherapy started).

The DD subgroup was the only group that consistently demonstrated a significant decrease of both MDSC subsets up until post-therapy completion (*p* = 0.0078 for PMN-MDSC and *p* = 0.0078 for M-MDSC). Meanwhile, any significant changes of MDSCs level in the other subgroups of patients were not observable. Interestingly, the DD subgroup also had a significantly higher M-MDSCs baseline level compared to the other subgroup (*p* = 0.0238 vs DI subgroup and *p* = 0.0314 vs M subgroup) (Appendix Table A3).

Analysis on the association between tumor status and MDSCs response was also investigated. In terms of tumor staging, although the circulating MDSCs subset at baseline level were found to be slightly higher in the metastatic group; however, the Pearson test resulted in insignificant association with the MDSCs profile based on overall or stratified-group analysis. Similar insignificant association were also observed between circulating MDSC levels with RAS mutation based on overall analysis (Appendix Table A5).

**Fig. 4:**
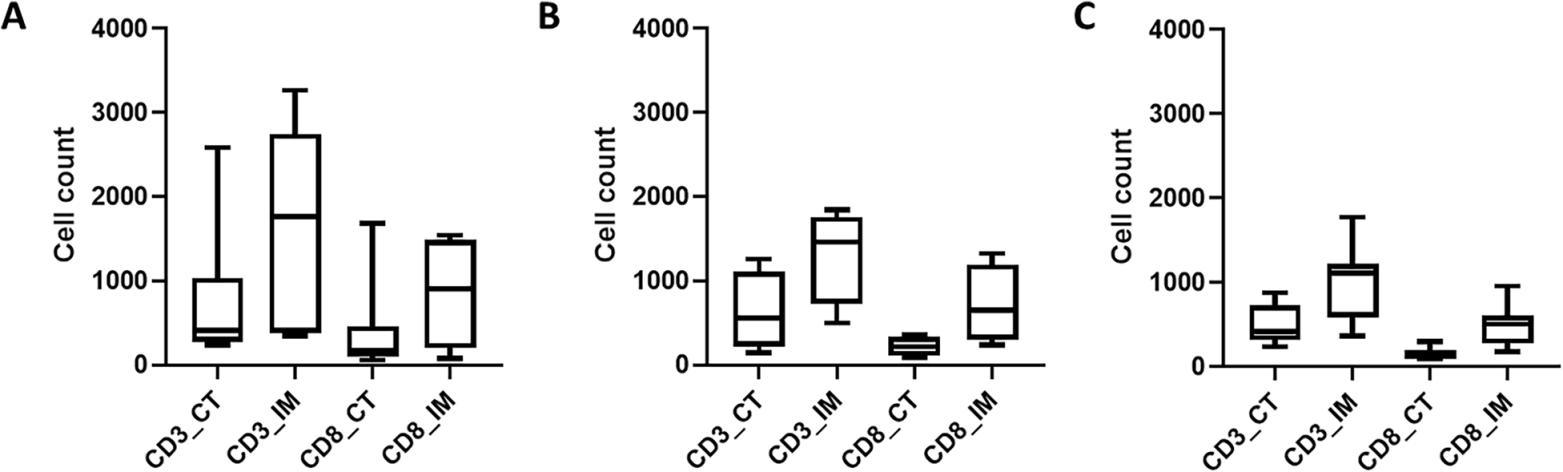
TILs profiles based on D-14-MDSCs response stratification. TILs profile for double decreased (DD) group (**A**), TILs profile for double increased group (DI) (**B**), and TILs profile for mixed group (M) (**C**)

In addition, we also assessed the density of TILs in tumor. As described in Fig. 4, although it was not statistically significant, we observed a slightly higher median value of cells density within tumor invasive margin of DD subgroup (1758 for CD3 + cells and 903 for CD8 + cells, respectively) compared to DI subgroup (1463 for CD3 + cells and 650 for CD8 + cells) that was relatively similar with the M subgroup (1114 for CD3 + cells and 508 for CD8 + cells).

**Fig. 5:**
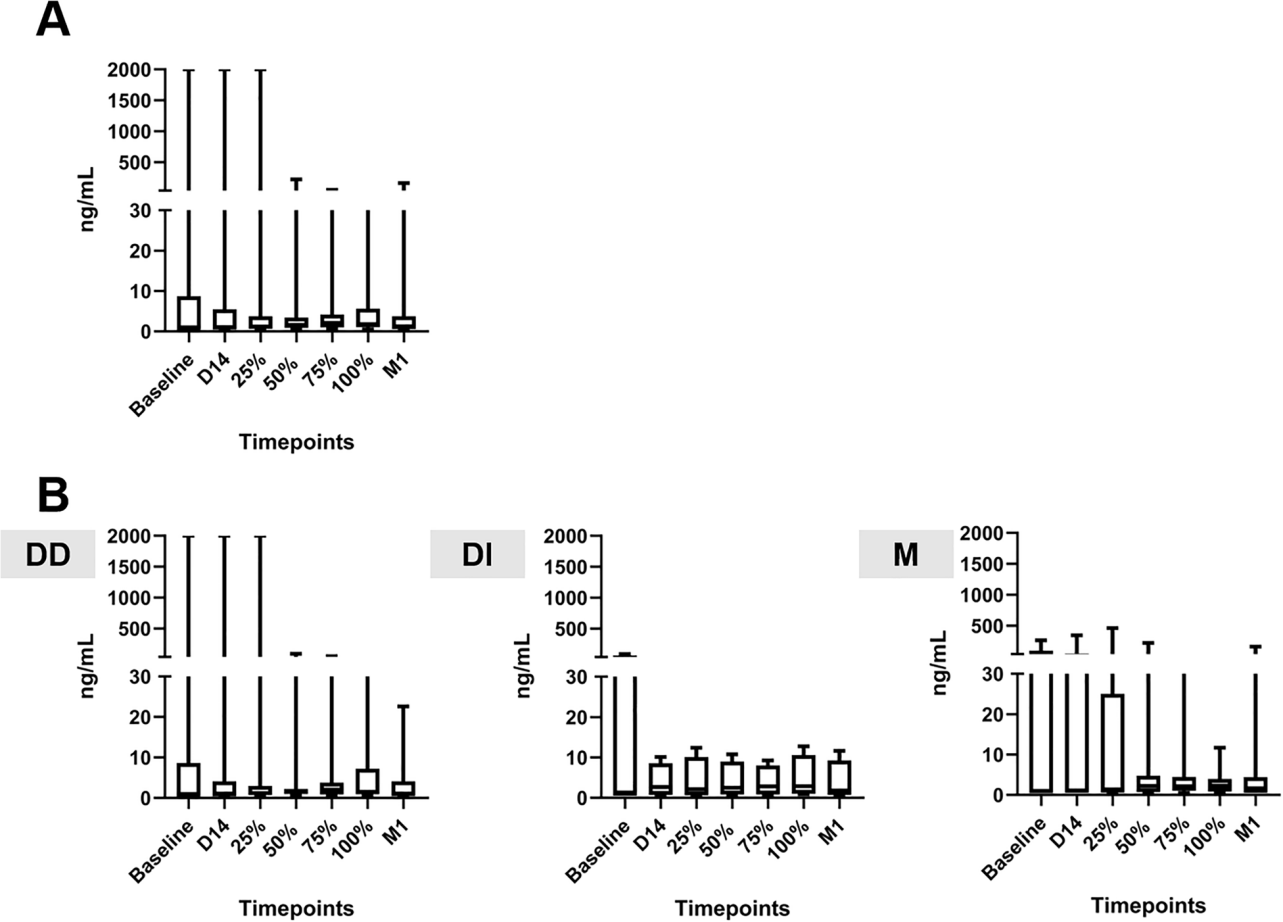
CEA profile based on overall analysis (**A**) and stratified to D-14 MDSCs response (**B**). The data represented as median of CEA level in ng/mL blood. DD = double decreased, meaning there was a decreased level of MDSCs in both PMN- and M-MDSCs at D-14; DI = double increased, meaning there was an increased level of MDSCs in both PMN- and M-MDSCs at D-14; M = increased/decreased level of PMN- and M-MDSCs at D-14 in a mixed manner

In parallel, the CEA level was also assessed at all time points of sampling to observe the response pattern and to explore its potential correlation with MDSC response. As seen in Fig. 5, both the overall groups showed insignificant changes in CEA levels during the study. Interestingly, we observed that the DD subgroup tended to have a more stable or decreasing CEA level compared to the DI and M subgroups which had relatively similar pattern with the MDSC level during the study.

## Discussion

It is widely accepted that the level of circulating MDSCs increases in the advanced stage of cancer, correlating with disease progression and formation of metastases [5]. Numerous studies have also shown that several chemotherapeutic agents in conventional cancer chemotherapy modulated MDSCs numbers in tumor tissues as well as in the peripheral lymphoid organs thus implicating the treatment outcome [12, 14]. In the present study, we documented the profile of circulating MDSCs in a cohort of treatment-naive CRC patients and how oxaliplatin-based chemotherapy shaped this circulating MDSCs level during and after treatment completion. Our study results supported previous studies that the circulating MDSC level was increased significantly in CRC patients compared to the healthy subjects. Furthermore, our study showed that only a subgroup of CRC patients with decreased of both circulating PMN- and M-MDSCs levels on D-14 (*n* = 9) of treatment consistently demonstrated this significant decrease of MDSC level during and after therapy completion compared to baseline (*p* = 0.031).

Despite there is no consensus regarding its phenotypic classification, MDSC is commonly subdivided into granulocytic PMN-MDSC and monocytic M-MDSC based on expression of CD14 and CD15 cell surface markers [9, 10, 15]. Most studies agreed that MDSCs presence were increased in PBMC of cancer patients when compared to healthy donors [9, 18, 19]. However, there are slight discrepancies between studies regarding subset profiles. Some studies reported that both M- and PMN-MDSCs were significantly more accumulated in the blood of colon cancer patients [19] and head and neck cancer patients [20] than in healthy donors. Meanwhile, others found that only the PMN-MDSC subset expanded significantly in the blood of colon cancer patients [14] as well as other cancer patients [18]. This discrepancy might be influenced by many factors. Several authors proposed that a technical approach during blood preparation prior to flow cytometry assessment [9, 20] and different cancer types and staging [8] or types of markers used to identify MDSC subsets [20] could influence the result. Considering this issue, our study used whole blood as our initial samples in order to minimize any impacts of sample processing and to maintain MDSC profile similar to in vivo conditions [11]. In addition, we implemented markers such as CD33 and HLA-DR as surface biomarkers for MDSCs identification then followed by CD15 for PMN-MDSCs, or CD14 for M-MDSCs during flow cytometry analysis according to previous studies [14, 18]. Therefore, consistent with those studies, this technical approach revealed that only the PMN-MDSCs subset was increased significantly in CRC patients while the M-MDSCs subset remained insignificant.

Our study showed that the MDSCs subset was present at a low proportion in the peripheral blood of healthy subjects and it significantly increased in the CRC patients. Similar results were observed in another study with circulating MDSC levels at 1.89% ± 0.75% in CRC patients and 0.54% ± 0.35% indicating a low percentage of CD33 HLA DR subset in peripheral blood [21]. In addition, despite all statuses were confirmed as treatment-naive CRC patients, wide variations of MDSCs level were still observed between patients. Previous study proposed multiple factors might influence the circulating MDSCs level, including tumor stage, the time interval prior to chemotherapy, and resection history of patients [9, 22]. These factors may have influenced both the baseline measurements and final outcomes, raising concerns that our protocol might overlook the effects of initial tumor removal on MDSCs dynamics. Since such data were not obtained, all these aspects are limitations of our study. However, this study captured the dynamic profile of MDSCs during chemotherapy in real-world conditions reflecting the inherent aspect of patient heterogeneity in clinical practice.

Combinations of chemotherapeutic agents that are commonly used to target cancer cells have also been reported to regulate MDSC-mediated immunosuppression in cancer patients [23, 24]. One of the most widely used first-line anticancer drugs for CRC, oxaliplatin, can potentially deplete MDSCs with minimal effect on effector T cells [25]. Studies in both in vitro and in vivo in tumor-bearing mice showed that oxaliplatin could deplete and promote MDSCs maturation which later inhibited their immunosuppressive behavior [25]. Administration of even a low dose of oxaliplatin could lower the expression of ARG1 and (NADPH Oxidase 2) NOX2 in MDSCs inhibiting its suppressive function. Lastly, oxaliplatin has also been reported to modulate MDSCs suppressive ability via the downregulation of NF-κB signaling activation [25]. Oxaliplatin in combination with fluorouracil and folinic acid (FOLFOX) is known to be one of the main strategies for patients with advanced-stage CRC [26, 27]. FOLFOX therapy on metastatic CRC patients could significantly decrease MDSCs level in peripheral blood allowing the effective antitumor medication. Therefore, the elimination of MDSC should be considered an auxiliary therapeutic target in CRC therapy management since their presence contributes to tumor progression.

In this study, we found that oxaliplatin-based chemotherapy reduced the MDSC levels preferentially in a subgroup of patients who demonstrated a significant decrease of both MDSC subsets at D-14 after chemotherapy administration (DD subgroup) while other subgroups (DI and M) of patients demonstrated relatively stable level during the study. Furthermore, we observed that this DD subgroup was likely to have higher circulating PMN-MDSC levels compared to other subgroups (Appendix Table A3). In line with our finding, previous study also found a similar phenomenon [14]. They observed a combination of FOLFOX-bevacizumab induced depletion in PMN-MDSC subset preferentially in patients with initial high levels of PMN-MDSCs (≥ 1% within PBMC) and raised a hypothesis that PMN-MDSCs are more sensitive to the chemotherapy-induced cell death effect thus led to a significant decrease of their level following chemotherapy initiation.

There is also an interesting result found in the TILs profile between subgroups. Patients belong to the DD subgroup were more likely to have higher CD3 and CD8 T cell numbers within their invasive margins compared to the other subgroups. Previous studies suggested that increased MDSCs can negatively impact the infiltration and activity of TILs by inhibiting T cell activation, reducing the production of T cell-activating cytokines, and promoting the generation of regulatory T cells [28, 29]. Our study showed that the DD subgroup consistently demonstrated a declining circulating MDSCs trend, which might indicate a similar condition in the tumor site. Ideally, further study comparing the MDSCs between TME and peripheral blood would provide more accurate data to support the hypothesis. However, due to its complication and technical challenges on MDSCs characterization within the tumor site, such as the small population of MDSCs and complexity of distinguishing MDSCs with other phenotypically and morphologically similar myeloid cells [30], the study in human subjects is difficult to achieve. Combining several markers could possibly be done to overcome this challenge; however, the best marker combinations that allow a clear distinction between MDSCs and other myeloid cells are yet to be discovered. Therefore, characterizing MDSCs using freshly isolated cells remains the most appropriate [31]. Alternatively, another study suggested that LOX-1 + receptor biomarker presented by PMN cells in peripheral blood can be used as potential additional biomarker since they demonstrated suppressive activity against T cell proliferation in tumor site [32].

In conjunction with the immune aspect represented by the MDSC level described previously, this study also evaluated other tumor biomarker aspects to confirm the DD subgroup profile in comparison with other subgroups. In terms of CEA level, in likewise previous study, we observed a slight increase of CEA level following chemotherapy administration in all patient subgroups [33, 34]; however, the changes of CEA level remain insignificant between those subgroups (Appendix Table A4). Other tumor biomarkers such as KRAS mutation and BRAF mutation also did not show any significant different between subgroups. Besides, it is worth noting that our study did not provide clinical outcome data thus the association between MDSCs profile, especially of the DD subgroup, needs to be further investigated.

## Conclusion

In conclusion, our study showed that circulating MDSCs level, particularly the PMN-MDSCs subset in CRC patients was significantly higher compared to healthy subjects. Oxaliplatin-based therapy might have a beneficial effect by reducing MDSC level in a subgroup of CRC patients who showed both decreased PMN- and M-MDSC levels at day 14 after chemotherapy administration. Further investigation on CRC patients with more homogenous clinical baseline status and evaluation of both circulating and TME MDSCs remains necessary to support these findings.

## Supplementary Information

Below is the link to the electronic supplementary material.

Supplementary file1 (DOCX 590 KB)

## Data Availability

No datasets were generated or analysed during the current study.
