# Supplementary material for: The Dynamic Changes of Circulating Myeloid-Derived Suppressor Cells (MDSCs) Subsets in Colorectal Cancer Patients Undergoing Oxaliplatin-Based Chemotherapy
Source: J Gastrointest Cancer. 2025 Mar 26;56(1):87. doi: 10.1007/s12029-025-01207-x (PMC11947042; doi:10.1007/s12029-025-01207-x)
Supplement: Supplementary file 1 — Supplementary file1 (DOCX 590 KB) [file 12029_2025_1207_MOESM1_ESM.docx]

**APPENDIX**


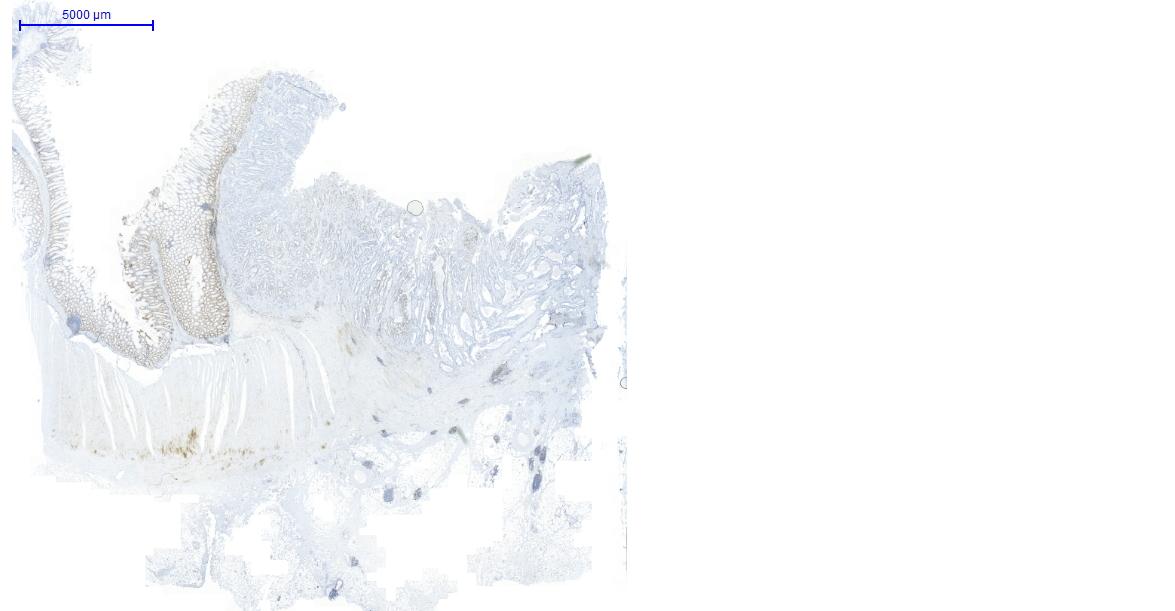


**CT**

**IM**

**Fig A1**. Visualization of tumor tissue profiling area to assess tumor infiltrating lymphocytes (TILs). The assessment requires tissue area at least 1.5 – 2 centimeters in size including invasive margin (IM) and center of tumor (CT) as marked in the figure.


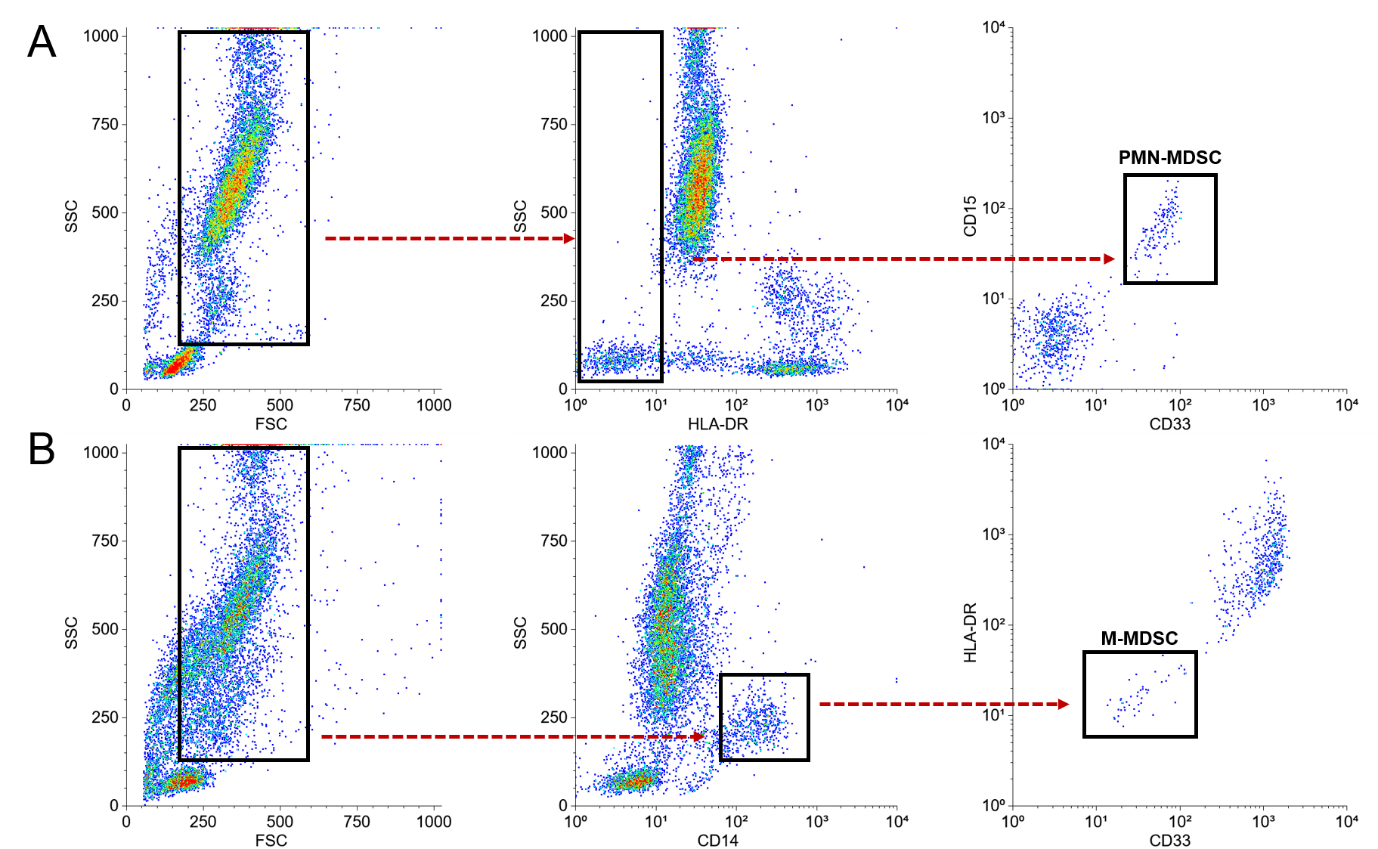


**Fig A2**. Gating strategy for MDSCs analysis within peripheral blood for subpopulation PMN-MDSC (A) and M-MDSC (B)

***Appendix Table A1****. Summary of circulating MDSCs on CRC patients vs healthy subjects*

|  | **PMN-MDSC** | | | **M-MDSC** | | |
| --- | --- | --- | --- | --- | --- | --- |
| Subjects | Median | Interquartile Range | P-value | Median | Interquartile Range | P-value |
| Healthy subjects (n=14) | 0.08 | 0.19 | 0.003 | 0.26 | 0.97 | 0.890 |
| CRC patients (n=30) | 0.28 | 0.29 |  | 0.22 | 0.49 |  |

***Appendix Table A2****. Summary of circulating MDSCs level data on all subjects*

|  |  | PMN-MDSC (n=21) | | | M-MDSC (n=21) | | |
| --- | --- | --- | --- | --- | --- | --- | --- |
|  | Time Points | Median | Interquartile Range | P-value  (vs Baseline) | Median | Interquartile Range | P-value  (vs Baseline) |
|  | Baseline | 0.30 | 0.270 | N/A | 0.17 | 0.495 | N/A |
|  | D14 | 0.13 | 0.210 | 0.0975 | 0.09 | 0.400 | 0.1774 |
|  | 25% | 0.09 | 0.095 | 0.0002 | 0.17 | 0.440 | 0.9295 |
|  | 50% | 0.11 | 0.185 | 0.0062 | 0.16 | 0.475 | 0.9451 |
|  | 75% | 0.15 | 0.115 | 0.0024 | 0.17 | 0.225 | 0.6149 |
|  | 100% | 0.13 | 0.225 | 0.0080 | 0.10 | 0.250 | 0.5346 |
|  | M1 | 0.14 | 0.215 | 0.0145 | 0.08 | 0.345 | 0.2214 |

|  |  | Both subsets Decreased (DD, n=9) | | | Both subsets Increased (DI, n=4) | | | Mixed (M, n=8) | | | P-value  (Between groups) | |
| --- | --- | --- | --- | --- | --- | --- | --- | --- | --- | --- | --- | --- |
|  | Time Points | Median | Interquartile Range | P-value  (vs Baseline) | Median | Interquartile Range | P-value  (vs Baseline) | Median | Interquartile Range | P-value  (vs Baseline) | DD vs DI | DD vs M |
| PMN-MDSC (%) | Baseline | 0.37 | 0.23 | N/A | 0.075 | 0.6975 | N/A | 0.220 | 0.2900 | N/A | 0.1566 | 0.1919 |
|  | D14 | 0.10 | 0.10 | 0.0039 | 0.165 | 0.4875 | 0.875 | 0.280 | 0.3275 | 0.9453 | 0.3301 | 0.2078 |
|  | 25% | 0.13 | 0.07 | 0.0117 | 0.075 | 0.0400 | >0,9999 | 0.070 | 0.1000 | 0.0156 | 0.0294 | 0.2442 |
|  | 50% | 0.11 | 0.22 | 0.0195 | 0.215 | 0.4475 | 0.875 | 0.090 | 0.1375 | 0.1094 | 0.6042 | 0.8333 |
|  | 75% | 0.15 | 0.22 | 0.0273 | 0.045 | 0.0775 | 0.625 | 0.140 | 0.0975 | 0.1484 | 0.0196 | 0.4342 |
|  | 100% | 0.15 | 0.18 | 0.0117 | 0.030 | 0.0425 | 0.625 | 0.120 | 0.3000 | 0.3828 | 0.0112 | 0.9807 |
|  | M1 | 0.12 | 0.16 | 0.0078 | 0.135 | 0.4125 | 0.875 | 0.205 | 0.2650 | 0.6641 | 0.6839 | 0.2858 |

***Appendix Table A3****. Summary of circulating MDSCs level data on stratified subjects*

|  |  | Both subsets Decreased (DD, n=9) | | | Both subsets Increased (DI, n=4) | | | Mixed (M, n=8) | | | P-value  (Between groups) | |
| --- | --- | --- | --- | --- | --- | --- | --- | --- | --- | --- | --- | --- |
|  | Time Points | Median | Interquartile Range | P-value  (vs Baseline) | Median | Interquartile Range | P-value  (vs Baseline) | Median | Interquartile Range | P-value  (vs Baseline) | DD vs DI | DD vs M |
| M-MDSC (%) | Baseline | 0.34 | 1.96 | N/A | 0.075 | 0.2025 | N/A | 0.000 | 0.2675 | N/A | 0.0238 | 0.0314 |
|  | D14 | 0.11 | 0.40 | 0.0039 | 0.305 | 2.2005 | 0.125 | 0.000 | 0.0875 | >0,9999 | 0.2559 | 0.2030 |
|  | 25% | 0.14 | 0.73 | 0.2500 | 0.215 | 1.1175 | 0.250 | 0.250 | 0.4650 | 0.4063 | 0.5007 | >0,9999 |
|  | 50% | 0.19 | 0.65 | 0.1563 | 0.415 | 1.3375 | 0.250 | 0.150 | 0.2675 | 0.5000 | 0.7091 | 0.3517 |
|  | 75% | 0.22 | 0.92 | 0.3125 | 0.105 | 0.0925 | 0.875 | 0.115 | 0.2500 | 0.9063 | 0.1399 | 0.1585 |
|  | 100% | 0.29 | 0.57 | 0.3008 | 0.125 | 0.1950 | 0.125 | 0.030 | 0.2425 | >0,9999 | 0.1385 | 0.0478 |
|  | M1 | 0.14 | 0.28 | 0.0078 | 0.320 | 0.6775 | 0.625 | 0.040 | 0.3225 | 0.8438 | 0.7986 | 0.9499 |

***Appendix Table A4****. Summary of serum CEA level data on stratified subjects*

|  |  | Both subsets Decreased (DD, n=9) | | | Both subsets Increased (DI, n=4) | | | Mixed (M, n=8) | | | P-value  (Between groups) | |
| --- | --- | --- | --- | --- | --- | --- | --- | --- | --- | --- | --- | --- |
|  | Time Points | Median | Interquartile Range | P-value  (vs Baseline) | Median | Interquartile Range | P-value  (vs Baseline) | Median | Interquartile Range | P-value  (vs Baseline) | DD vs DI | DD vs M |
| CEA (ng/mL) | Baseline | 0.76 | 8.125 | N/A | 1.300 | 65.8850 | N/A | 0.955 | 97.7700 | N/A | 0.8028 | 0.9870 |
|  | D14 | 0.91 | 3.48 | >0,9999 | 2.695 | 7.7880 | >0,9999 | 1.110 | 47.7700 | 0.3125 | 0.5007 | 0.8731 |
|  | 25% | 1.23 | 2.29 | 0.8125 | 2.150 | 9.5000 | >0,9999 | 1.370 | 24.5300 | 0.3125 | 0.9720 | >0,9999 |
|  | 50% | 1.57 | 1.26 | 0.9102 | 2.510 | 8.1375 | 0.8750 | 2.230 | 4.0675 | 0.9375 | 0.8252 | 0.8148 |
|  | 75% | 2.07 | 2.88 | 0.9453 | 2.850 | 7.1450 | 0.8750 | 2.155 | 3.3900 | 0.9375 | 0.7105 | 0.7989 |
|  | 100% | 1.45 | 6.15 | 0.8203 | 2.885 | 9.6050 | 0.8750 | 2.280 | 3.1380 | 0.9375 | 0.8252 | 0.9626 |
|  | M1 | 0.96 | 3.52 | 0.6875 | 1.820 | 8.5375 | >0,9999 | 1.655 | 3.8650 | >0,9999 | 0.5007 | 0.6557 |

***Appendix Table A5****. Summary of circulating MDSC level on patients with RAS mutation and RAS wild type.*

|  |  | RAS Mutant (n=11) | | | RAS Wild type (n=10) | | |
| --- | --- | --- | --- | --- | --- | --- | --- |
|  | Time Points | Median | Interquartile Range | P-value  (vs baseline) | Median | Interquartile Range | P-value  (vs baseline) |
| PMN-MDSC (%) | Baseline | 0.300 | 0.47 | N/A | 0.300 | 0.2475 | N/A |
|  | D14 | 0.120 | 0.13 | 0.3652 | 0.165 | 0.2250 | 0.1309 |
|  | 25% | 0.090 | 0.06 | 0.0098 | 0.145 | 0.1625 | 0.0156 |
|  | 50% | 0.190 | 0.22 | 0.0977 | 0.080 | 0.1500 | 0.0391 |
|  | 75% | 0.150 | 0.22 | 0.0869 | 0.115 | 0.1175 | 0.0098 |
|  | 100% | 0.050 | 0.11 | 0.0137 | 0.160 | 0.2350 | 0.2754 |
|  | M1 | 0.150 | 0.23 | 0.0400 | 0.130 | 0.2075 | 0.2090 |
| M-MDSC (%) | Baseline | 0.220 | 0.65 | N/A | 0.170 | 0.9825 | N/A |
|  | D14 | 0.390 | 0.50 | >0,9999 | 0.040 | 0.095 | 0.0508 |
|  | 25% | 0.260 | 0.47 | 0.7002 | 0.120 | 0.4325 | 0.7422 |
|  | 50% | 0.150 | 0.74 | 0.9297 | 0.165 | 0.2925 | 0.7871 |
|  | 75% | 0.130 | 0.21 | 0.5625 | 0.195 | 0.2675 | >0,9999 |
|  | 100% | 0.100 | 0.22 | 0.1855 | 0.230 | 0.655 | 0.8203 |
|  | M1 | 0.140 | 0.64 | 0.3223 | 0.04 | 0.265 | 0.4805 |
